# 10 tips on how to use dynamic risk assessment and alerts for AKI

**DOI:** 10.1093/ckj/sfae325

**Published:** 2024-10-29

**Authors:** Danica Quickfall, Ashley M La, Jay L Koyner

**Affiliations:** Committee on Clinical Pharmacology and Pharmacogenomics, Biological Science Division, University of Chicago, Chicago, IL, USA; Section of Nephrology, Department of Medicine, University of Chicago, Chicago, IL, USA; Committee on Clinical Pharmacology and Pharmacogenomics, Biological Science Division, University of Chicago, Chicago, IL, USA; Section of Nephrology, Department of Medicine, University of Chicago, Chicago, IL, USA

**Keywords:** acute kidney injury, alerts, biomarkers, clinical decision support, renal replacement therapy

## Abstract

Acute kidney injury (AKI) is a common syndrome in hospitalized patients and is associated with increased morbidity and mortality. The focus of AKI care requires a shift away from strictly supportive management of established injury to the early identification and timely prevention of worsening renal injury. Identifying patients at risk for developing or progression of severe AKI is crucial for improving patient outcomes, reducing the length of hospitalization and minimizing resource utilization. Implementation of dynamic risk scores and incorporation of novel biomarkers show promise for early detection and minimizing progression of AKI. Like any risk assessment tools, these require further external validation in a variety of clinical settings prior to widespread implementation. Additionally, alerts that may minimize exposure to a variety of nephrotoxic medications or prompt early nephrology consultation are shown to reduce the incidence and progression of AKI severity and enhance renal recovery. While dynamic risk scores and alerts are valuable, implementation requires thoughtfulness and should be used in conjunction with the overall clinical picture in certain situations, particularly when considering the initiation of fluid and diuretic administration or renal replacement therapy. Despite the contemporary challenges encountered with alert fatigue, implementing an alert-based bundle to improve AKI care is associated with improved outcomes, even when implementation is incomplete. Lastly, all alert-based interventions should be validated at an institutional level and assessed for their ability to improve institutionally relevant and clinically meaningful outcomes, reduce resource utilization and provide cost-effective interventions.

Acute kidney injury (AKI) is broadly defined as an abrupt (over hours to days) decrease in kidney function, with the current consensus definition, the Kidney Disease: Improving Global Outcomes (KDIGO) defining AKI based on changes in either serum creatinine and/or urine output [1]. AKI is a very common clinical syndrome that can occur in 10–20% of hospitalized patients and up to 30–40% of critically ill patients. AKI is consistently associated with increased morbidity and mortality regardless of its clinical source or clinical location. Most AKI-focused care is supportive rather than aimed at treating the underlying source of AKI, but past data demonstrate that many patients with AKI do not get appropriate care [2, 3].

Given this increased risk of adverse outcomes combined with clear gaps in clinical care, there have been several investigations around early AKI detection via biomarkers, alerts and clinical decision support systems (CDSSs) to help improve the implementation of guideline-based AKI care. This review seeks to delineate the existing literature around these alerts as well as highlight the process around implementing an alert or CDSS. These processes and the outcome measures are different across clinical settings, with alerts targeting patients at high risk for severe AKI by identifying them through novel biomarkers or the presence of Stage 1 AKI [4, 5] being very different from those seeking to reduce the nephrotoxin burden on patients at risk for or with AKI [6, 7]. Alerts may be passive, notifying the team of the AKI (or AKI risk), while others use a more active process linking the alert with a CDSS or a care bundle to guide interventions with the hopes of improving patient outcomes (Fig. 1). The investigations into the clinical utility of alerts and CDSSs have been mixed, with inconsistent results across a variety of clinical settings, making their interpretation difficult, if not confusing. We seek to review this growing field and provide some clarity on the strengths and weakness of alerts and CDSSs in the setting of increased AKI risk as well as established AKI.

**Figure 1:**
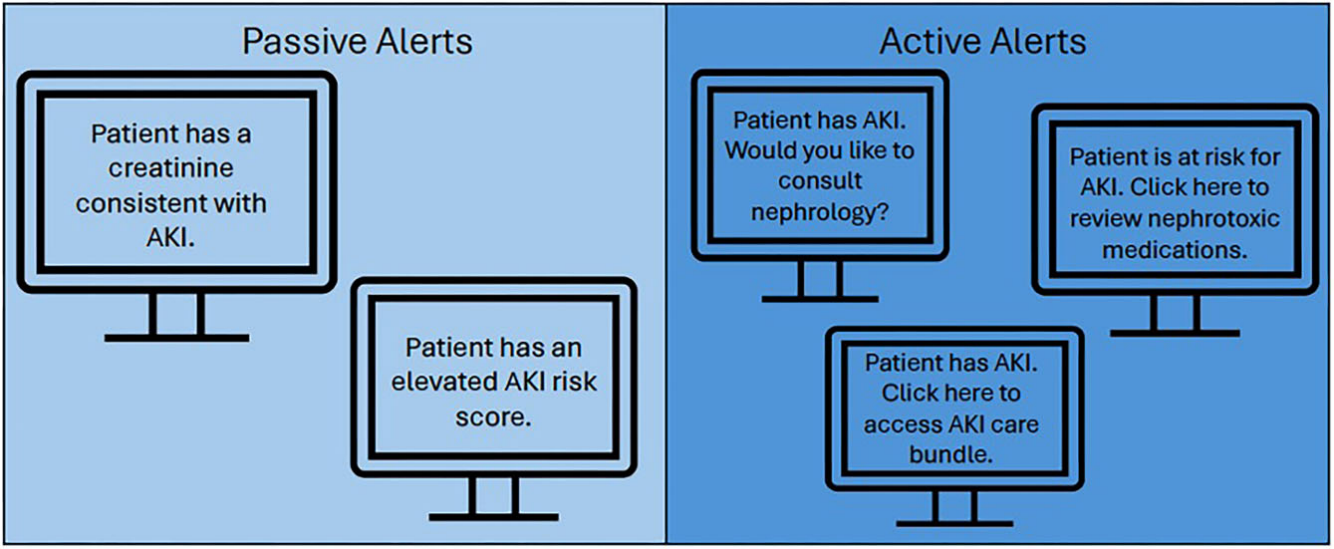
Passive versus active alerts. The figure demonstrates examples of passive and active alert systems implemented in electronic health systems.

## EXTERNAL AND REPEATED VALIDATION OF ACCURATE DYNAMIC AKI RISK ASSESSMENT TOOLS IS NEEDED

Historically, AKI risk scores have been static, assessing risk at a singular moment in time [e.g. prior to cardiac surgery or at intensive care unit (ICU) arrival] [8–11]. Many of these scores, while externally validated, still leave room for improved risk stratification. More recently, models have moved from static to dynamic, with more scores adding data that are amassed across an admission rather that at a single moment in time. Several studies have demonstrated that using intra- and postoperative data improves the ability to predict the development of postoperative AKI [12, 13]. Similarly, other risk scores have demonstrated that risk assessment improves by adding data from day 1 or day 2 of an ICU stay [14, 15]. Other risk scores have used advanced learning techniques and artificial intelligence (AI) to estimate AKI risk across all hospitalized patients and demonstrated areas under the receiver operating characteristics curve >0.90 in predicting Stage 2 and 3 AKI or the need for dialysis [16, 17]. We anticipate that there will be more and more risk scores developed over the next several years, however, to date, not all of these scores have been externally validated. The validation of these risk scores is important, as there is growing literature demonstrating that well-developed models do not perform the same way across all centres [18].

**Figure 2:**
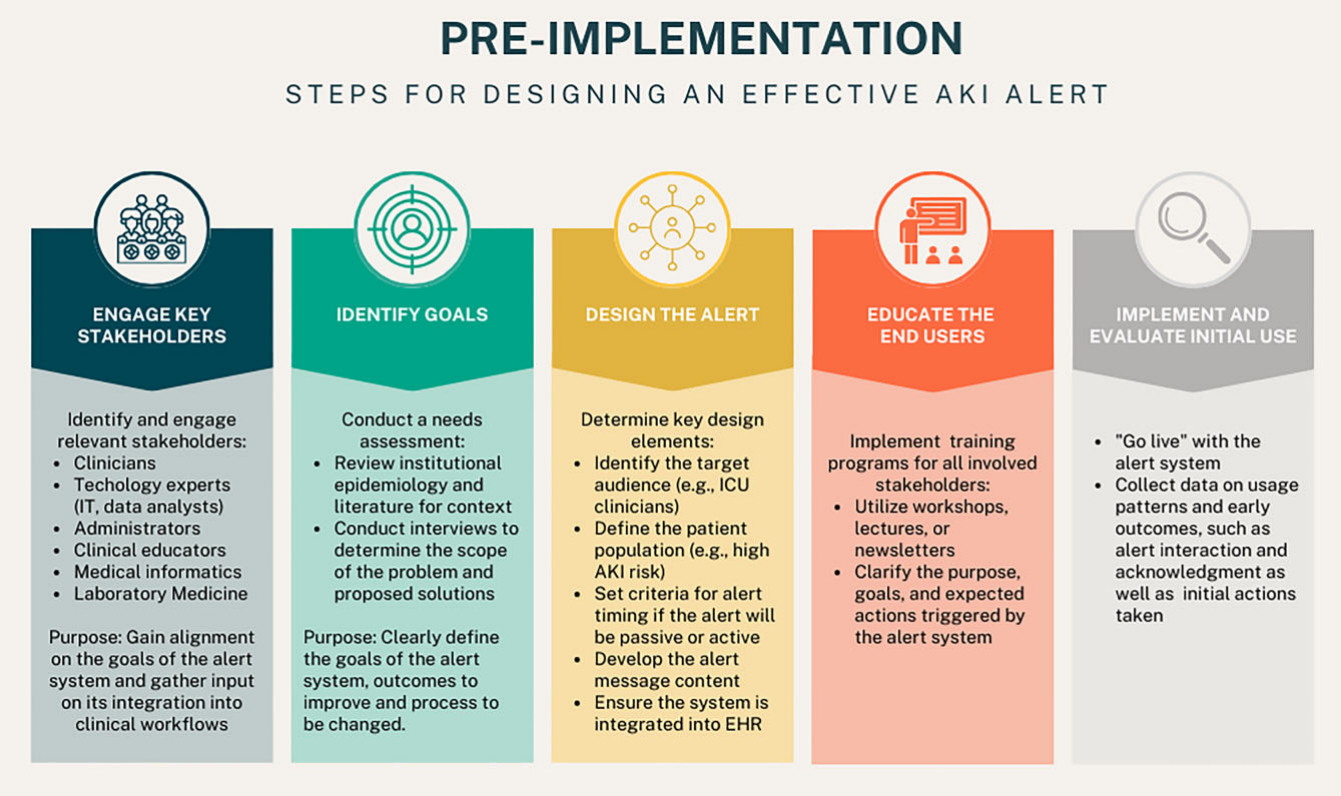
Step prior to implementation of a risk score or alert. Figure provides several steps throughout the process of designing and implementing an alert. Start with engaging all stakeholders to ensure that the goals of the alert are clear to all (clinical and non-clinical). Once the goals are aligned, the alert needs to be designed, tested and all stakeholders need to be educated around the alert.

Figure 2 demonstrates the steps prior to the implementation of a clinical risk score/AKI alert. Prior to the clinical implementation of any risk score it is important to understand what is needed to set up, validate and maintain any risk scores, as well as understanding their potential weaknesses. For example, not all scores include information from prior to the index hospitalization, and while risk scores in the setting of elective cardiac surgery routinely account for baseline kidney function, this is not always available in the setting of critical illness or emergent hospitalization, thus understanding the impact and potential confounding of known or unknown pre-admission kidney function is important when evaluating an AKI risk score. Separately, ensuring that these complex dynamic scores are providing reliable, accurate risk assessment is no small task. Quality assurance and vigilance around persistent score performance is crucial, as upgrades to the electronic medical record, errors in data entry and changes in the way care is delivered over time may all impact the accuracy of a given score/threshold. However, before any dynamic risk score is ready for widespread implementation it must first demonstrate the ability to improve AKI patient outcomes.

## ALERTS TO PREDICT AKI RISK ARE ENHANCED BY BIOMARKERS OF EARLY RENAL INJURY

Many alert systems utilize risk scores that involve a combination of comorbidities, vital signs and laboratory data to predict the risk of AKI [12, 14]. While risk scores incorporate many risk factors for AKI, the renal-specific components of risk scores are often limited to serum creatinine (SCr), but significant injury to the kidney can occur before any increase in creatinine, limiting the ability to predict AKI early [19, 20]. Novel urinary biomarkers have been developed with the goal of predicting AKI earlier than SCr or urine output, with some urinary biomarkers capable of distinguishing specific types of AKI, such as acute tubular necrosis, hepatorenal syndrome and acute interstitial nephritis [21–24].

Another goal of identifying novel biomarkers to predict AKI earlier than SCr increase is to prevent AKI. Tissue inhibitor of metalloproteinase-2 (TIMP-2) and insulin-like growth factor binding protein 7 (IGFBP7) are both proteins that are involved in G1 cell cycle arrest and are released by renal tubular cells in response to cellular stress, and the combination of these two proteins in the urine has been validated for predicting the impending development of stage 2 or 3 AKI in critically ill patients [25]. Thus elevated urinary TIMP-2*IGFBP7 is associated with an increased risk of AKI, with a high-sensitivity cut-off of 0.3 (ng/ml)^2^/1000 and a high-specificity cut-off of 2.0 (ng/ml)^2^/1000. Table 1 summarizes data regarding biomarkers to predict AKI and their role in AKI care.

**Table 1:** Novel biomarkers used to predict AKI risk.

Biomarker	Outcome	Cut-off	Clinical application
Urinary TIMP-2*IGFBP7 [73]	Predict stage 2 or 3 AKI in critically ill patients	>0.3 (ng/ml)^2^/1000 (high sensitivity) >2 (ng/ml)^2^/1000 (high specificity)	PrevAKI RCT [26]: Elevated TIMP-2*IGFBP7 linked to care bundle in post-cardiac surgery patients reduced the incidence of AKI PrevAKI-multicenter RCT [74]: Elevated TIMP-2*IGFBP7 linked to care bundle in post-cardiac surgery patients improved care bundle adherence and reduced the incidence of stage 2/3 AKI BigpAK RCT [27]: Elevated TIMP-2*IGFBP7 linked to care bundle in post-non-cardiac surgery patients reduced the incidence of stage 2/3 AKI
Urinary NGAL [75]	Predict stage 2 or 3 AKI in critically ill patients	>78.0 ng/ml	TAKING FOCUS 2 [27]: Protocol utilizing AKI risk score, urinary NGAL and furosemide stress test to guide fluid management improved survival to paediatric ICU discharge

Subsequent to validating the biomarker to predict AKI, several studies have shown benefits of implementing kidney-focused care bundles in response to elevated urinary TIMP-2*IGFBP7. One such study was the PrevAKI trial, which randomized cardiac surgery patients with a postoperative TIMP-2*IGFBP7 level ≥0.3 (ng/ml)^2^/1000 to receive standard care or a strict care bundle involving invasive haemodynamic monitoring with a prespecified algorithm for pressure and volume management, discontinuation of nephrotoxins and avoidance of hyperglycaemia [26]. The PrevAKI trial found that the intervention group had reduced rates of AKI (55% versus 72%; *P* = .004), although it was not powered to assess differences in renal replacement therapy (RRT) or mortality (*P* = NS for both) [26]. Similarly, the BigpAK study randomized non-cardiac surgery patients with an elevated postoperative TIMP-2*IGFBP7 to standard care or a CDSS care bundle [27]. This study found that both the incidence of stage 2 or 3 AKI (7% versus 20%; *P* = .04) and length of hospital stay (16 days versus 21 days; *P* = .04) were reduced in the intervention group [27]. While the BigpAK study is currently undergoing large-scale validation [28], collectively these studies demonstrate how biomarker values can prompt the use of kidney-focused care bundles for patients at risk for AKI.

While the PrevAKI and BigpAK trials demonstrate the potential clinical utility of TIMP-2*IGFBP7, there are inherent factors in these trials that limit their generalizability. Both trials included specific surgical populations, and randomization to the intervention group meant strict adherence to a prespecified care protocol. Beyond these specific patient populations and outside of a trial setting, adherence to care bundles may be less successful, such as in medically complex critically ill patients where treatment must be individualized rather than protocolized. For example, it may be unreasonable to discontinue broad-spectrum nephrotoxic antibiotics despite the presence of AKI in a patient with refractory septic shock. A quality improvement study linking a care bundle to elevated TIMP-2*IGFBP7 suggested improvement in outcomes in a mixed critical care population when certain aspects of the care bundle were utilized, specifically early nephrology consultation [29].

Novel biomarkers may enhance AKI risk scores and prediction models, but this needs further investigation. Unlike SCr, biomarkers are not yet routinely utilized in all clinical settings, and clinicians must be trained on the proper clinical scenarios in which biomarker testing may be useful. For instance, TIMP2*IGFBP7 is less accurate in the setting of nephrotic-range proteinuria or advanced liver disease with significant hyperbilirubinuria [30]. Perhaps an alert system can first identify patients who may benefit from biomarker measurement, then subsequent risk scores utilizing the biomarker can alert clinicians to elevated AKI risk. Finally, risk scores should be paired with a clinically appropriate care bundle. After further validation studies, there may be a role for using novel biomarkers in alerts and risk scores to trigger specific interventions for AKI prevention.

## CDSSs AROUND NEPHROTOXINS CAN REDUCE THE INCIDENCE OF AKI

The development of AKI and subsequent progression to severe AKI may be preventable using early alert-based identification and mitigation strategies [31]. Approximately one of every four in-hospital kidney injuries is the result of nephrotoxic medication exposure [32]. Medications can affect kidney function through a variety of mechanisms, including but not limited to changes in glomerular haemodynamics, inflammation, tubular toxicity and crystal nephropathy.

A single-centre quality improvement initiative conducted in non-critically ill paediatric patients demonstrated nephrotoxic medication exposure was reduced by 38% and AKI rates were reduced by 64% when practitioners received a medication-based CDSS alert in patients receiving medications associated with increased AKI risk [33]. In this quality improvement project, clinicians received an alert with advice to measure SCr daily and consider using an alternative medication when multiple nephrotoxic medications were prescribed. The findings of this study were subsequently validated across nine paediatric centres with a sustained 23% reduction in AKI [7]. An attempt to model a similar medication-based CDSS in all hospitalized adults was effective at reducing medication exposure, however, it did not demonstrate a significant effect on progression of AKI, dialysis or death. This difference may reflect the inclusion of a more vulnerable, comorbid adult population where SCr is often measured daily, the types of medications included in alerts, which may have variable rates of AKI across the age spectrum, or the inclusion of critically ill adult patients who are at higher risk of adverse outcomes compared with ward-based paediatric patients. While some exposure to nephrotoxic medications is unavoidable, increased vigilance through daily creatinine monitoring, switching to a less toxic alternative medication or stopping nephrotoxic medications when possible were all effectively achieved using medication alert–based interventions.

## AUTOMATED ALERTS IDENTIFY THOSE WHO WILL BENEFIT FROM NEPHROLOGY CONSULTATION

In many locations, nephrology consultation is both a scarce resource and a key component in the care of patients with AKI, providing individualized prevention and treatment regimens across a variety of clinical settings. Patients for which nephrology is consulted tend to have more comorbidities, higher severity of acute illness and more advanced AKI than patients without a nephrology consult [29, 34, 35]. While several retrospective investigations have shown that early nephrology consultation within 48 hours of AKI is associated with reduced mortality and other improved outcomes [35–37], the interventions recommended and then subsequently implemented during consultation are highly varied and delayed consults and implementation allow for the accumulation of more tubular injury and adverse outcomes.

In one before-and-after study utilizing an active electronic health record (EHR) alert to notify clinicians of patients at risk for AKI or with stage 1 AKI in which one group (*n* = 7881) was randomized to have their team receive a care bundle link, the incidence of hospital-acquired AKI was slightly reduced after implementation of the alert {odds ratio [OR] 0.99 [95% confidence interval (CI) 0.98–1.00], *P* = .049}, and perhaps more importantly, mortality among patients who developed AKI was reduced (27% versus 22%; *P* = .021) [38]. The care bundle used in this study included considering discussion with nephrology if AKI does not improve at 24 hours. While analysis of process measures did not include differences in nephrology consultation, the authors did find that there was more discontinuation of potential nephrotoxins (*P* < .001) and more documentation of AKI (*P* = .033) in the care bundle arm [38].

In a study by Park *et al.* [39], an EHR alert notified clinicians when a patient's SCr met the definition of AKI and asked the clinician whether a nephrology consult was requested. In this before-and-after study, not only did the alert lead to a reduction in overlooked AKI (6% versus 18%; *P* < .001), but early consultation rates also increased (28% versus 6.5%; *P* < .001). This alert led to lower rates of severe AKI (27% versus 32%; *P* = .007) and higher rates of renal recovery (80% versus 56%; *P* < .001) [39]. As a before-and-after study, the improvement in AKI-related outcomes may be confounded by overall improvement in AKI care over time unrelated to nephrology consults; however, subgroup analysis demonstrated that patients with early nephrology consultation were more likely to recover renal function than those without consultation (81.9% versus 63.3%; *P* < .001), and perhaps the notification of AKI is key to reminding providers to enact appropriate AKI care. Another confounder in this before-and-after study is the Hawthorne effect, where providers may change their practice knowing that the EHR is able to track whether the CDSS recommendation was followed, especially if they chose not to consult nephrology. A limitation of this alert system was that AKI screening occurred only at midnight for next-morning alerts, which assisted information-processing efficiency and prevented alert fatigue throughout the day, but this likely caused a delay in nephrology consultation relative to AKI development and, as the authors pointed out, affected the mortality analysis (which was not significant) because most deaths occurred on the day of AKI.

While not all alert studies have shown a beneficial effect on AKI, it may be that passive alerts, those not linked to a bundle or a pre-specified action, are less likely to improve outcomes [4]. Both of these studies were performed in an academic medical centre, and different results may occur at non-teaching hospitals where there are fewer members of medical teams to recognize AKI and consultation practices may differ. Since early nephrology consults are associated with improved patient outcomes, alerts can provide timely assistance in identifying patients who may benefit from a nephrology consult. Further studies assessing the impact of AKI alerts triggering structured nephrology consults could be optimized using cluster randomized study designs in order to minimize temporal effects on care and should investigate effects in various hospital settings.

It is important to acknowledge that given the high prevalence of hospital-acquired AKI, it would be impossible for a nephrologist to provide care for every patient with AKI, let alone at risk for AKI. Risk scores and alerts can provide guidance in triaging which patients would benefit most from the limited resource of nephrology consultation. Perhaps in the future the process can be more automated. As additional novel AKI biomarkers become validated and incorporated into risk scores, biomarkers may provide prognostic insight into which patients are likely to progress to or sustain severe AKI and identify those who would benefit from kidney-focused care. Future studies should investigate whether nephrology consults improve outcomes of patients with elevated levels of various biomarkers.

## VARIABLE OUTCOMES ASSOCIATED WITH USING ALERTS FOR FLUID MANAGEMENT

One of the hallmarks of AKI care is fluid balance assessment and correction (including resuscitation, de-escalation and the avoidance and treatment of volume overload). Many studies and applications of EHR alerts for fluid administration focus on patients with sepsis/septic shock, where fluid balance has been shown to impact mortality [40, 41]. In one study investigating the effects of a text alert for patients identified by a computer prediction tool as having sepsis, the proportion of patients who received intravenous fluids within 12 hours was increased in the intervention group compared with the control group (38% versus 24%; *P* = .013) [42]. Additionally, in a before-and-after study from Hayden *et al.* [43] utilizing an EHR alert for at-risk sepsis patients, time to fluid bolus was reduced post-implementation by 31 minutes (51 minutes versus 82 minutes; *P* < .01). While patients with chronic kidney disease (CKD) and end-stage renal disease (ESRD) are more likely to receive conservative fluid resuscitation rather than the standard 30 ml/kg bolus, it has been shown that patients, even those with ESRD, who do not receive a 30 ml/kg fluid bolus within 3 hours of the onset of sepsis have a higher mortality rate [44, 45]. Alerts can provide reminders for early fluid resuscitation in patients with sepsis, including for patients with CKD or ESRD.

In terms of AKI, one active alert system (linked to fluid order sets) identifying patients with SCr-based AKI resulted in increased fluid administration (23% versus 5%; *P* < .01) and diuretic use (4% versus 3%; *P* < .01) post-implementation, as well as more AKI resolution (66% versus 61%; *P* = .048) [46]. However, not all showed a benefit in AKI and fluid administration, as a pooled analysis of multiple studies investigating the effects of electronic alerts on AKI management found no differences in fluid administration [47]. One explanation for the inconsistent results related to fluid administration is because AKI is a heterogeneous condition and the indication for fluids is often nuanced. For example, patients with hepatorenal or cardiorenal syndrome would not benefit and may actually be harmed by fluid administration and the AKI phenotype (and volume status) is not always readily available on clinical presentation. Thus fluid administration may not always demonstrate positive outcomes in AKI care in studies and the decision to give intravenous fluids should be individualized.

While fluid administration may benefit some patients with AKI, fluid overload is associated with increased mortality among critically ill patients, especially among patients with AKI [48, 49]. Investigation into optimal methods to identify patients who would benefit from deresuscitation is ongoing, and thus there are few studies investigating the use of alerts for fluid overload. Akcan-Arikan *et al.* [50] developed a real-time kidney injury score that incorporated fluid overload when cumulative fluid balance was >15% of paediatric ICU admission weight. Higher scores were associated with higher rates of mortality and longer lengths of stay [50]. In another study of critically ill paediatric patients, a combination of a risk score, urinary neutrophil gelatinase-associated lipocalin (NGAL) and furosemide stress test for urine output was used to guide fluid management and continuous renal replacement therapy (CRRT) initiation [51]. NGAL is a protein that is upregulated in damaged renal tubule cells to stimulate tubule re-epithelialization, and elevated levels in the urine have been used as an early biomarker for AKI, specifically in the paediatric population [51, 52]. Implementation of this multimodal protocol resulted in lower fluid accumulation (4% versus 12%; *P* < .01) and a higher rate of survival to ICU discharge (65% versus 46%; *P* = .02) [51]. A confounding factor in this study is that NGAL is also largely expressed by neutrophils and may be elevated in infections independent of AKI. Nonetheless, critically ill patients with infections are at high risk for AKI and fluid overload, and preventing excessive fluid accumulation in both populations is important. This study was also limited to paediatric ICU patients, but ongoing studies are validating the use of urinary NGAL in AKI risk prediction scores and CDSS alerts in adult ICU patients. While these studies do not specifically alert clinicians to fluid overload, they suggest that fluid balance data can be incorporated into risk scores and protocols that could be used in future AKI alerts to improve patient outcomes.

Unfortunately, the use of real-time kidney injury scores has not been assessed in adult patients or across a variety of disease states, including burn, trauma and surgical settings. As a result, the thresholds for which adults experience the worst outcomes it is not known and the benchmarks for reducing fluid accumulation to improve outcomes are not yet part of consensus guidelines. Based on the success of these interventions in paediatric studies, these should be further evaluated in adults across a variety of ICU settings.

## REDUCING ADMINISTRATION OF PROTON PUMP INHIBITORS (PPI) IS ASSOCIATED WITH IMPROVED CLINICAL OUTCOMES

PPIs are associated with a wide range of adverse events, including increased risk of infection, osteoporosis and development of both AKI and CKD [53]. Several plausible mechanisms for the development of kidney-related adverse outcomes exist, but the most frequently described is a hypersensitivity reaction leading to the development of acute/chronic interstitial nephritis [54]. While the cornerstone of drug-induced AKI management involves withdrawal of offending medications, there are currently no international guidelines that address discontinuation of PPIs in patients with AKI.

Wilson *et al.* [6] conducted a multicentre study aimed at reducing nephrotoxic drug exposure in hospitalized adults with established AKI. They examined the impact of AKI alerts on both the rate of medication discontinuation and the development of a composite outcome including progression of AKI, receipt of RRT or death within 14 days. This study evaluated alerts for patients with AKI receiving one of three different classes of medications: non-steroidal anti-inflammatory drugs (NSAIDs), renin–angiotensin–aldosterone system inhibitors (RAASis) and PPIs. This EHR-based alert study did not find any improvement in composite outcomes for those patients receiving NSAIDs or RAASis; however, the PPI-exposed subgroup analysis showed a statistically significant reduction in composite outcomes [relative risk 0.88 (95% CI 0.79–0.98), *P* = .02]. Unfortunately, only 22% of PPIs were discontinued when practitioners received an alert versus 17% discontinuation in the usual care group, while there were much higher rates of discontinuation in the NSAID and RAASi groups (60–80%) [6]. In the setting of AKI, discontinuing PPIs is associated with improved patient outcomes, and future EHR-based alerts should focus on increasing the discontinuation rates.

One of the major challenges posed by medication-based alerts is the opportunity for practitioner override without critical appraisal of the alert itself. This study by Wilson *et al.* [6] demonstrated a very low rate of PPI discontinuation, which is likely owing to practitioner's perception of the benign nature of PPIs compared with NSAID and RAASi medications. Additionally, it does not account for those in whom PPIs are imminently clinically indicated, e.g. admission with gastrointestinal bleeding or treatment of *Helicobacter pylori* infection. To truly assess the efficacy of these alert-based interventions on outcomes, these factors must be incorporated. Clinically relevant prescribing information as well as a critical analysis of the need for intervention should be considered when creating alerts aimed at reducing medication use.

## DYNAMIC CREATININE MONITORING MAY IDENTIFY VANCOMYCIN-ASSOCIATED AKI (VA-AKI)

Vancomycin is a frequently administered antibiotic in hospitalized patients that is primarily used to treat Gram-positive infections, including methicillin-resistant *Staphylococcus aureus*. Recently, the presence of vancomycin-associated tubular casts in biopsy specimens was described as a hallmark of vancomycin-induced nephrotoxicity [55]. The presence of tubular casts is particularly problematic because vancomycin is almost exclusively renally cleared. Impaired renal clearance leads to prolonged elevations in vancomycin levels and further amplifies renal toxicity [56]. While there are no established vancomycin level cutoffs leading to toxicity, some studies have described an incidence of AKI of ≈30–40% in those achieving a vancomycin area under the curve/minimum inhibitory concentration (AUC/MIC) >500–600 mg/h/l [57, 58]. These levels correspond to the high end of accepted therapeutic targets [59].

An unusually steep increase in SCr has been reported in patients with suspected VA-AKI [60]. An early mean increase in SCr of 260 μmol/l (2.6 mg/dl) was observed in patients with VA-AKI compared with an increase of 88–132 μmol/l (1–1.5 mg/dl) in patients with other causes of severe oligoanuric AKI, including those with non-vancomycin drug-induced kidney injury [60]. The sharp increase in creatinine typically occurred within 2 days, compared with 5–7 days described in other drug-induced kidney injuries [61]. Early daily monitoring of SCr in patients receiving vancomycin may be helpful in detecting VA-AKI and help differentiate it from other aetiologies.

While there have been no specific dynamic creatinine-based EHR alerts for vancomycin reported in the literature, this idea of alerting providers about those with large changes in SCr and receiving vancomycin provides an example of an automated dynamic creatinine alert to discontinue prescribing, switch to a non-nephrotoxic alternative or reduce the dose early in the course of VA-AKI to minimize nephrotoxicity.

## ALERT-BASED SYSTEMS ARE NOT A SUBSTITUTE FOR CLINICAL DECISION-MAKING AND SHOULD NOT BE USED TO INITIATE RRT

Given its role in defining AKI, many EHR alerts incorporate both absolute and dynamic changes in SCr levels. Unfortunately, since creatinine is generally a marker of glomerular filtration, in critically ill patients SCr levels do not necessarily reflect the degree of kidney tubular damage. It was previously demonstrated that patients with a higher SCr level at the time of RRT initiation had improved outcomes and overall survival [62]. This may reflect that lower creatinine levels are associated with significant volume overload, poor nutritional status and low muscle mass at baseline or a loss of mass during critical illness in those with poor outcomes and that high SCr concentrations may reflect increased muscle mass and indicate better overall baseline health.

The timing of RRT has been a topic of much debate in the literature. Studies previously found conflicting effects on overall survival and long-term dependence on RRT when comparing early versus delayed initiation of RRT [63–65]. While the triggers used in recent trials investigating the initiation of RRT were variable, several trials randomized patients with KDIGO stage 2 or 3 AKI to early initiation of RRT. In comparison, delayed cohorts required prespecified triggers to initiate CRRT, including clinical evidence of volume overload, persistent or refractory metabolic complications (including acidosis or hyperkalaemia) and persistent severe AKI after 48–72 hours. In most of these trials, including the largest [STARRT-AKI (NCT02568722)] trial, there was no observed mortality benefit associated with early initiation of RRT and there was a signal for harm, with early initiation being linked to a higher 90-day dependence on RRT and an increased incidence of adverse events (hypotension and hypophosphataemia). It should be noted that <15% of those who were screened for STARRT-AKI were eventually enrolled, so clinicians need to be mindful about misinterpreting these findings and applying them to all comers [66]. As a result, when appropriate, the decision to initiate RRT in critically ill patients should be dictated by the presence of volume overload, metabolic complications or persistent severe AKI as clinically indicated, and not on the basis of SCr (alerts) alone. In the future, creation of more comprehensive alerts may help refine the timing of RRT initiation. The use of AI-based algorithms that incorporate patient factors including comorbidities, disease processes and more subtle changes in volume status, acid–base balance and metabolic changes in addition to novel biomarkers and changes in SCr should be explored to provide a more personalized approach to CRRT in critically ill patients.

## INDIVIDUALIZE ALERTS TO INSTITUTIONS BY CONTINUOUSLY MEASURING RESOURCE UTILIZATION AND OUTCOMES

EHR alerts assist in patient care and improve outcomes, especially alerts that have specific goals and specific guidance. However, as several of the aforementioned studies have shown, there is variability in their impact on outcomes. The success of an alert system depends on the communication culture, patient populations, resource accessibility and other factors that can vary by institution. To ensure an alert system is functioning as intended, it is important to regularly assess practice patterns and outcome measures after implementation of a new alert system, as well as to adapt alert systems to the evolving needs of the institution (Fig. 2).

The impact of an implemented alert system should include healthcare spending and resource utilization. This ensures that the alerts are changing practice and that the cost of the alerts is outweighed by improved patient outcomes (and cost savings from preventing AKI itself). A multicentre stepped wedge cluster randomized trial by Selby *et al.* [67] demonstrated that their AKI alert system increased AKI recognition (89% versus 69%; *P* < .001) and led to a shorter duration of AKI by nearly 1 day (*P* = .01). These positive findings occurred with no significant increase in resource utilization (including nephrology consultation and renal ultrasound), suggesting a favourable cost–benefit balance. In contrast, in a separate randomized trial utilizing an AKI alert system [4], renal consults among surgical patients increased in the alert group (11.5% versus 5%; *P* = .006), but there were no differences in AKI severity or mortality, suggesting a less favourable cost–benefit balance. It is unclear why there was higher resource utilization without complementary improved patient outcomes, such as whether effects were attenuated because providers were more attentive to AKI and care guidelines for patients randomized to usual care (no alert), or whether this is a false positive finding in a subgroup analysis. Regardless, what works in one centre may not work in another, thus individual institutions should assess cost–benefit balances, and revisions may involve adjusting the alert criteria and/or method or editing the recommendations provided with the alert.

Lastly, it is essential to ensure that an alert system is not causing harm. In a multicentre trial, patients with AKI were randomized to an EHR pop-up notifying providers of AKI with links to an AKI management order set [68]. In assessing the outcomes associated with implementing the alert, it was found that while there was no difference in mortality between the alert and usual care groups in teaching hospitals, the alert was associated with a higher risk of death in non-teaching hospitals (16% versus 9%; *P* = .003), although it was unclear through secondary analyses why this signal for harm was specific to non-teaching hospitals, as increased risk was linked to the use of intravenous fluids and diuretics [68]. Since AKI is a heterogeneous disease, a therapy that may help one patient with AKI may harm a different patient with AKI, and these differences may not be captured in measured data. Furthermore, some important systems factors, such as communication practices, are also difficult to measure. Thus it is essential for individual institutions to assess and optimize alert systems based on the real-time needs of that institution.

## AKI ALERTS NEED TO BALANCE ALERT FATIGUE WITH ALERT COMPLETION

Alert fatigue occurs when clinicians are exposed to an excessive number of alerts, leading to an increased risk of potentially ignoring a clinically relevant alert. Poor alert design and the sheer number of alerts can contribute to fatigue [69]. Regardless of the reason for alert fatigue, it must be avoided, as several studies found low event rates of bundle completion while others demonstrate that even partial response to an alert or bundle leads to improved outcomes [70–72]. In an investigation of an AKI-based alert paired with a care bundle, Kolhe *et al*. [71] demonstrated that outcomes correlate with completion of the alert bundle. Hospitalized patient mortality was lowest (18%) in those who had completion of their alert bundle within 24 hours. There was a stepwise increase in mortality in those who had their bundle completed in >24 hours, those who had partial completion and those who had no portion of the bundle completed. This effect persisted when looking at 30- and 60-day mortality. Similarly in other studies, certain bundle components were shown to have a greater impact on improving outcome [70]. Future investigators should focus future efforts on determining which care bundle elements are most valuable in specific AKI phenotypes, with subsequent prospective trials demonstrating that completion of those key elements will optimize patient outcomes while simultaneously limiting alert fatigue.

## CONCLUSION

**Figure 3:**
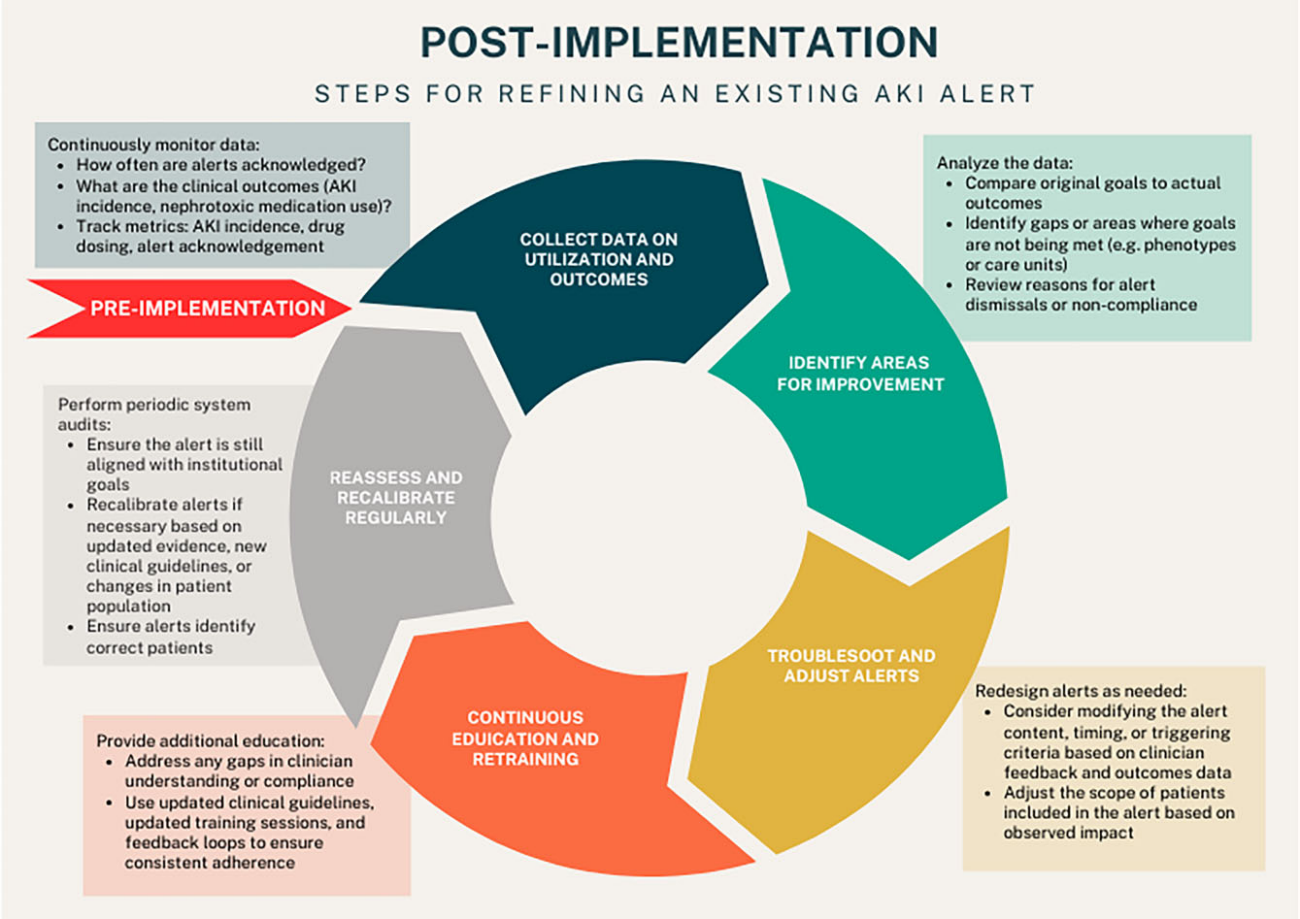
The post-implementation life cycle of an AKI alert. The figure demonstrates the process of refinement of an AKI alert following implementation (red arrow). Start with the determination of outcomes (before and after the alert) and then identify areas for improvement and work to troubleshoot and adjust the alert as feasible or needed. If changes are made, stakeholders should be educated and the process of recalibration and retesting the alert should occur, then iterative process can happen again.

Recent years have seen several studies investigating both passive and active alerts and CDSSs in the setting of clinical AKI (or increased AKI risk). While active alerts/CDSSs are more frequently associated with improved patient outcomes versus passive alerts, the results remain variable depending on the study and clinical setting. Importantly, most active AKI alerts are linked with some form of improved outcome (more guideline-based care, less severe AKI, shorter length of stay, decreased mortality). While nephrotoxin-based interventions have been slightly more successful than those focused on other areas of AKI, more studies are needed and prior studies still need large-scale validation. If an alert is successful in the setting of sepsis-associated AKI there is no guarantee it will also work in cardiac surgery-associated AKI, and as such it should be re-investigated. Once alerts are clinically implemented, they still require maintenance to ensure they remain clinically appropriate and associated with sustained improvements in patient care and/or outcome. Figure 3 demonstrates the processes that need to occur after the implementation of a risk score or alert. This requires the continued vigilance of nephrologists and all the other AKI stakeholders throughout the hospital.

## Data Availability

No new data were generated or analysed in support of this research.
